# Long Noncoding RNA LIT3527 Knockdown induces Apoptosis and Autophagy through inhibiting mTOR pathway in Gastric Cancer Cells

**DOI:** 10.7150/jca.58185

**Published:** 2021-06-11

**Authors:** Boya Wang, Yujie Deng, Juan Jin, Yan Wu, Lirong Shen

**Affiliations:** 1 Department of pharmacy, Sir Run Run Shaw Hospital, Zhejiang University School of Medicine, Hangzhou 310020, China.; 2 Institute of Gastroenterology, Zhejiang University, Hangzhou 310016, China.; 3 Department of Cell Biology, Zhejiang University School of Medicine, Hangzhou 310058, China.

**Keywords:** lncRNA, stomach, cell cycle, cell survival, metastasis, mTOR

## Abstract

Gastric cancer is one of the most common cancers and the leading causes of cancer mortality. However, the molecular mechanisms of gastric cancer malignancy remain unclear. Long noncoding RNAs (lncRNAs) have been well documented in controlling cancer progression. Identification of critical lncRNAs in gastric cancer will provide new sights into the regulation mechanism of gastric cancer. Here, we screened differentially expressed lncRNAs in gastric cancer tissues and matched adjacent tissues and found that lncRNA LIT3527, a 486-nucleotide (nt) sense transcript, was frequently upregulated in gastric cancer tissues. Knockdown of LIT3527 dramatically suppressed proliferation and migration of gastric cancer cells through inducing severe cell death but not affecting cell cycle. Mechanistically, we uncovered that depletion of LIT35227 induced significant cell apoptosis and autophagy through inhibiting AKT/ERK/mTOR signaling pathway. Targeting LIT3527 showed a robust inhibition of lung metastasis of gastric cancer cells. Taken together, these results suggest that LIT3527 is essential for gastric cancer cell survival through maintaining mTOR activity, suggesting that it may be clinically valuable as a therapeutic target for gastric cancer.

## Introduction

Gastric cancer is one of the most common malignant tumors and the second leading cause of cancer mortality worldwide 1. There were about one million cases that were diagnosed with gastric cancer worldwide each year, most of which occurred in East Asia 2. Although clinical researchers have made great progress in the treatment of gastric cancer, including surgical excision, new adjuvant chemo-radiotherapy, gene-targeted therapy and other comprehensive therapies, the overall survival time of gastric cancer patients remains unfavorable 3. Due to the lack of effective therapy, successful clinical management of metastatic GC remains a major challenge. Therefore, there is an urgent need to elucidate the molecular mechanism of gastric cancer progression and find novel diagnosis markers and therapeutic targets for gastric cancer patients.

Long noncoding RNAs (lncRNAs) are transcripts with more than 200 nucleotides in length and are unlikely to be translated to proteins 4. Numerous evidences have reported that lncRNAs play important roles in a variety of biological process, including chromatin modifications, transcriptional regulation, post transcriptional and other modes of gene expression regulation, and signal pathway regulation 5. It is well documented that aberrant expressions of lncRNAs are correlated with various types of cancer 6-8. Emerging roles of lncRNAs in cancer are involved in tumor initiation, apoptosis, immune escape, metabolic disorders, cancer cell stemness and metastasis 9.

More and more evidences also supported the critical roles of lncRNAs in gastric cancer malignancy 10. LncRNA GMAN is a crucial metastasis-related transcript for gastric cancer cells. GMAN promoted Ephrin A1 translational expression through competitively binding with its antisense GMAN-AS in a novel 'three RNA' model 11. LncRNA UCA1 upregulated PDL1 through inhibition of miR-26a/b, miR-193a and miR-214, leading to increased proliferation and migration and decreased apoptosis of gastric cancer cells 12. LncRNA HOXC-AS3 was activated by H3K4me3 and H3K27ac and upregulated in gastric cancer tissues. HOXC-AS3 interacted with YBX1 and modulated gene transcription in the tumorigenesis of gastric cancer 13. LncRNA GClnc1 acted as a modular scaffold to recruit WDR5 and KAT2A histone acetyltransferase complexes and facilitated transcription of target genes, and consequently enhanced gastric cancer cell proliferation and metastasis 14. These findings put a new insight into the roles of lncRNAs in gastric cancer progression. Therefore, identification of more critical lncRNAs will help us understand the molecular mechanisms underlying the tumorigenesis and malignancy of gastric cancer.

Here, we identified a new gastric cancer-related lncRNA LIT3527 from our lncRNA profiling dataset containing six human GC tissues and their matched non-tumor tissues. For the first time, we determined the critical roles of LIT3527 in maintaining cell survival of gastric cancer cells. Mechanistically, knockdown of LIT3527 induced apoptosis and autophagy of gastric cancer cells may through inhibiting the mammalian target of rapamycin (mTOR) pathway. The survival-related lncRNA LIT3527 might offer a good therapeutic target for gastric cancer treatment.

## Materials and Methods

### Human specimens and cell lines

All human tissue samples were obtained with informed consent from patients in the Sir Run Run Shaw Hospital, Zhejiang University School of Medicine (Hangzhou, China). Ethical consent was granted from the Ethical Committee Review Board of Zhejiang University School of Medicine.

AGS cells were obtained from the American Type Culture Collection (Manassas, VA, USA). MKN45, MKN74, HGC27, MGC-803 and GES-1 cells were obtained from The Cell Bank of Type Culture Collection of Chinese Academy of Sciences (Shanghai, China). These cells were maintained in RPMI 1640 with 10% FBS and antibiotics. BGC-823 cells from Institute of Biochemistry and Cell Biology, Chinese Academy of Sciences (Shanghai, China) 15 and SGC-7901 cells from Beijing Cancer Hospital (Beijing, China) 16 were maintained in Dulbecco's modified Eagle's medium (DMEM) supplemented with 10% fetal bovine serum (FBS, GIBCO, USA), penicillin/streptomycin. All cell lines were routinely tested for mycoplasma free.

### RNA extraction and quantitative RT-PCR

Total RNA was extracted from cell lines using TRIzol Reagent (Invitrogen, USA). Reverse transcription reactions were performed to produce cDNA using High-Capacity cDNA Reverse Transcription Kit (Applied Biosystems, USA). Quantitative real-time PCR (qRT-PCR) was carried out using the LightCycler 480 SYBR Green I Master (Roche, USA). qRT-PCR was performed in triplicate. Glyceraldehyde-3-phosphate dehydrogenase (GAPDH) was served as internal control. The primer sequences of indicated genes are as follows: *GAPDH*, sense 5'-GGACCTGACCTGCCGTCTAG-3', antisense 5'-TAGCCCAGGATGCCCTTAG-3'; *LIT3527*, sense 5'-GACTCTTGACCTATACTCTTAG-3', anti-sense 5'-TACGGGTCATAGGTTTCA-3'.

### siRNA and transfection

Gastric cancer cells were transfected with siRNAs using lifectamine^TM^ RNAiMAX (Invitrogen, USA) following the manufacture's protocol. The siRNAs were synthesized (GenePharma, China) and sequences are as follows: Control siRNA, UUCUCCGAACGUGUCACGUTT; LIT3527 siRNA1, 5'-CUCUGGCACUCAGAAUAAUTT-3'; LIT3527 siRNA2, 5'-GUUUAUGACUAAAUGGUGCTT-3'.

### Immunofluorescence

Cells were washed with ice cold PBS for three times, fixed in 4% paraformaldehyde at 4 °C for 1 h, and treated with 0.1% Triton X-100 at room temperature for 15 min. Then, cells were blocked with 5% BSA in PBS, incubated with antibodies against LC3 (1:500, Abcam, UK) overnight at 4 °C. After washing with PBST, cells were treated with Cy3-conjugated secondary antibodies (1:1000, Jackson ImmunoResearch, USA) for 1 h. DNA was stained with DAPI. The mounted coverslips were photographed by confocal fluorescence microscopy LSM510 (Zeiss, Germany).

### Immunoblot

Cells were lysed using RIPA protein extraction reagent (Beyotime, China) supplemented with a protease inhibitor cocktail (Roche, USA). Western blotting was carried out as previously reported 17. The proteins were detected using anti-LC3B antibody (Cell Signaling, USA), anti-4EBP1 antibody (Cell Signaling, USA), anti-mTOR antibody (Cell Signaling, USA), anti-p-mTOR antibody (Cell Signaling, USA), anti-Akt antibody (Diagbio, China), anti-p-Akt antibody (Diagbio, China), anti-Erk antibody (Diagbio, China), anti-p-Erk antibody (Diagbio, China), anti-GAPDH antibody (Sigma-Aldrich, USA) and corresponding secondary antibodies.

### Northern blot

Total RNAs from AGS cells or GES-1 cells were extracted with TRIzol Reagent (Invitrogen, USA). Northern blotting was carried out according to previous report 11. Briefly, 15 μg total RNA was separated on a 1.2% denaturing agarose gel with formaldehyde and transferred to Hybond N+ membrane (GE healthcare, USA). Digoxigenin-labeled RNA probes were prepared through *in vitro* transcription. The membrane was hybridized with denatured RNA probes at 60 °C for 16 hr, followed by incubation with anti-digoxigenin-AP (Roche, Switzerland). The results were analyzed using chemiluminescent imaging instrument (Clinx, China).

For *in vitro* transcription, LIT3527 was cloned into the pCS107 vector. Fluorescein-labeled RNA probe was transcribed from the linearized pCS107-LIT3527 plasmid using fluorescein RNA labeling mix (Roche, Switzerland), RNA inhibitor (Invitrogen, USA), and SP6 or T7 RNA polymerase (Roche, Switzerland) according to the manufacturer's instructions. The RNA probes were purified with RNeasy Mini Spin Column (Qiagen, Germany). The primer sequences for pCS107-LIT3527: Sense AGTCGGATCCTCAAGGATGACCGAGACGCC, antisense ACTGAAGCTTTGGGAATTGTGATCAGGGGA.

### Cell proliferation MTT assays

MTT (3-(4,5-dimethylthiazol-2-yl)-2,5-diphenyltetrazolium bromide) assays were performed at 24 h, 48 h, 72 h and 96 h post transfection. Cells with indicated transfection were seeded to 96-well plate with 3000 cells/well. At the indicated time point, cells were incubated with MTT (5 mg/ml, 20 μl) for 4 h at 37°C. Then discarded the medium, added 150 μl of dimethyl sulfoxide until the cysts were completely dissolved. The cell amount was analyzed by measuring the absorption at 490 nm with a spectrophotometer. On the other hand, at the indicated time point, the cultured cells were photographed under bright field. The cell number was counted from random six fields. Each assay was independently repeated three times.

### Transwell migration assay

Cells were harvested 36 h after transfection. Cells (5×10^4^ cells per well) were seeded to upper chambers of transwell inserts (8-μm pore, Corning, USA) in medium with 1% FBS. Medium supplemented with 10% FBS was added in the lower chamber. After incubation for 18 h, the migrated cells were stained with fluorescent dye 4'-6-Diamidino-2-phenylindole (DAPI, Sigma, USA) and quantified by counting in six random × 100 fields. Each assay was independently repeated three times.

### Scratch wound healing assay

Cells with indicated transfection were seeded in 24-well plates and allowed to grow at a density that reached 90% confluence. The monolayer cells were scratched with yellow pipette tips. Images were taken at 0 h, 12 h, 24 h after scratch. The wound healing distance was quantified by counting in five fields. Each assay was independently repeated three times.

### Cell cycle and apoptosis assays

For cell cycle analysis, cells were synchronized by serum starvation for 12 h and harvested at 48 h after transfection and fixed in ice-cold ethanol and stained with propidium iodide (Solarbio, China), then subjected to cell cycle analysis using FC 500 MCL Flow Cytometer (Beckman Coulter, USA). Cells were subjected to apoptosis analysis 60 h after transfection. Both adherent and floating cells were collected. Apoptosis assays were performed using Vybrant Apoptosis Assay Kit (Invitrogen, USA) with propidium iodide and fluorescein isothiocyanate (FITC)-conjugated annexin V. Each assay was independently repeated three times.

### Animal assay

The animal studies were approved by the Animal Care and Use Committee of Zhejiang University. Nude mice (5-6 weeks old) were cultured in SPF facilities, with individually ventilated cages and in a 12 h light-dark cycle with ad libitum access to food and water. For lung metastasis assay, we established *LIT3527*-depleted BGC-823 cells by lentivirus-based shRNAs. 10^6^ BGC-823 cells were inoculated into nude mice (n = 5) via tail-vein injection. After 5 weeks, the mice were sacrificed. The gross lungs were photographed and metastatic nodules were counted.

The lung tissues were fixed in 4% buffered formalin overnight. The fixed tissues were dehydrated in ethanol, embedded with paraffin and sectioned at 8 μm. For hematoxylin and eosin (H&E) staining, sections were stained with hematoxylin solution (Sigma, USA) and eosin solution (Sigma, USA) as previously described 18.

### Statistical analysis

Statistical analyses were performed using the GraphPad Prism 8 software. The intensity of immunoblot band was quantified by Image J software. Results were presented as the mean ± SD, and *P* < 0.05 was considered to be statistically significant.

## Results

### LncRNA LIT3527 is significantly upregulated in gastric cancer

**Figure 1:**
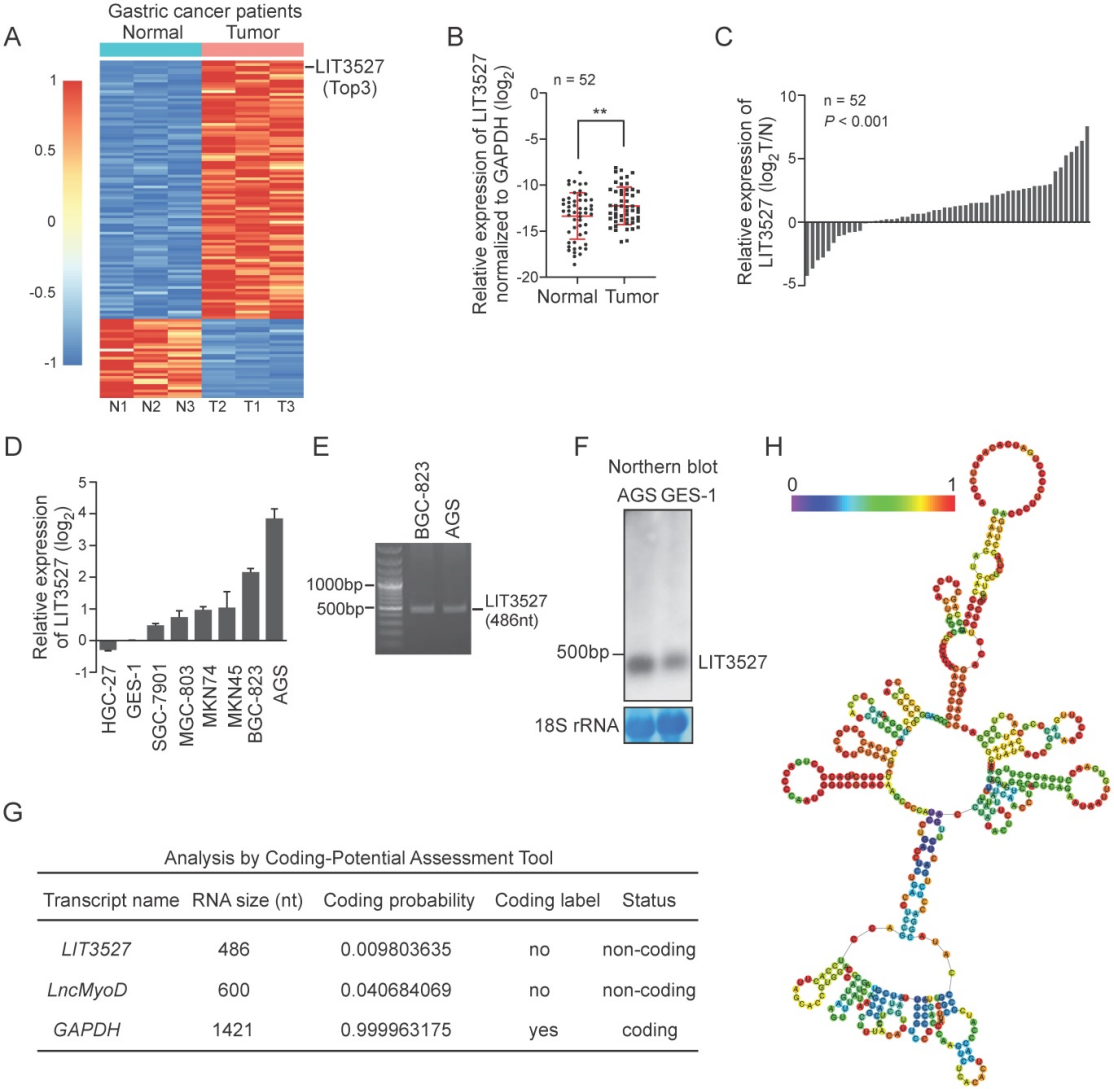
** LIT3527 is significantly upregulated in gastric cancer tissues. (A)** Hierarchical clustering showing differentially expressed lncRNAs in gastric cancer tissues and their matched non-tumor tissues from 3 male gastric cancer patients (fold change > 4 or fold change < 0.25, *P* < 0.01). 123 lncRNAs are upregulated, while 33 lncRNAs are downregulated in tumor tissues compared to matched non-tumor tissues. LIT3527 is one of the Top 3 upregulated lncRNAs in tumor tissues. **(B and C)** Quantitative RT-PCR analysis of LIT3527 expression in gastric cancer tissues and adjacent non-tumor tissues. Data are presented as log2 value of LIT3527 normalized to GAPDH (n = 52) (A). Waterfall plot showing the levels of LIT3527 in tumor tissues relative to adjacent non-tumor tissues (T/N) (B). **(D)** Relative levels of LIT3527 expression in human gastric cancer cell lines (AGS, BGC-823, MKN45, MKN74, MGC-803, SGC-7901, HGC-27) to the immortalized human gastric epithelial cell line (GES-1). **(E)** PCR result showing the length of LIT3527. LIT3527 is a transcript with 486 nt length. Total RNAs were extracted from AGS cells. **(F)** Northern blot analysis of LIT3527 expression in total RNAs from AGS cells and GES-1 cells. Methylene blue showing the 18S rRNA as a loading control. **(G)** The coding potential of *GMAN* predicted by Coding-Potential Assessment Tool. The well-known lncRNA *lncMyoD* and the protein-coding gene *GAPDH* are also shown. **(H)** The secondary structure of LIT3527 is predicted by 'RNAfold'. The graphic is minimum free energy structure drawing encoding base-pair probabilities. The color bar represents the base-pair probabilities. Data are shown as mean ± SD. **P < 0.01; paired *t* test (*B*), *t* test (*C*).

To study the critical lncRNAs in the gastric cancer progression, we analyzed the lncRNA profiling in the human gastric cancer tissues compared to matched non-tumor tissues (GSE106815). Since the incidence of gastric cancer was much higher in male than it in female, lncRNA profiling data from three male gastric cancer patients were analyzed to screen the differentially expressed lncRNAs. 123 significantly upregulated lncRNAs were identified in gastric cancer tissues (fold change > 4 or fold change < 0.5, *P* < 0.01) (Fig. 1A). Among the differentially expressed lncRNAs, lncRNA LIT3527 (GenBank access ID: AY927517) was one of the top 3 upregulated lncRNAs in gastric cancer tissues compared to matched non-tumor tissues (Fig. 1A). To confirm the lncRNA profiling results, we collected a gastric cancer cohort (n = 52) with 52 gastric cancer tumor tissues and matched non-tumor tissues. Quantitative RT-PCR (qRT-PCR) showed that LIT3527 was significantly upregulated in gastric cancer tissues compared to matched non-tumor tissues (Fig. 1B). Waterfall plot suggest LIT3527 was frequently upregulated in gastric cancer tissues (Fig. 1C). We further examined the LIT3527 expression in gastric cancer cell lines. Compared to GES-1, a normal gastric mucosa epithelial cell line, high expression of LIT3527 was observed in most of the gastric cancer cell lines, especially in AGS cells (Fig. 1D).

LIT3527 is a 486-nucleotide (nt) sense transcript. To study the gastric cancer related lncRNA LIT3527, we cloned the LIT3527 from gastric cancer cells (Fig. 1E). We designed a specific RNA probe for LIT3527 and it was detectable at its expected size in AGS cells and GES-1 cells by northern blot (Fig. 1F).

To understand the function of LIT3527 in gastric cancer, we first analyzed the coding potential of LIT3527 using Coding potential Assessment Tool algorithms 19. The well-known lncRNA *lncMyoD* and the protein-coding gene *GAPDH* were served as control. LIT3527 was predicted as non-coding RNA (Fig. 1G). We further analyzed the secondary structure of LIT3527. RNAfold predicted the secondary structure of LIT3527 according to minimum free energy with encoding base-pair probabilities 20. There are many loops in the secondary structure of LIT3527, suggesting the strong binding potential of LIT3527 with other molecules (Fig. 1H).

### LIT3527 is essential for gastric cancer cell proliferation

**Figure 2:**
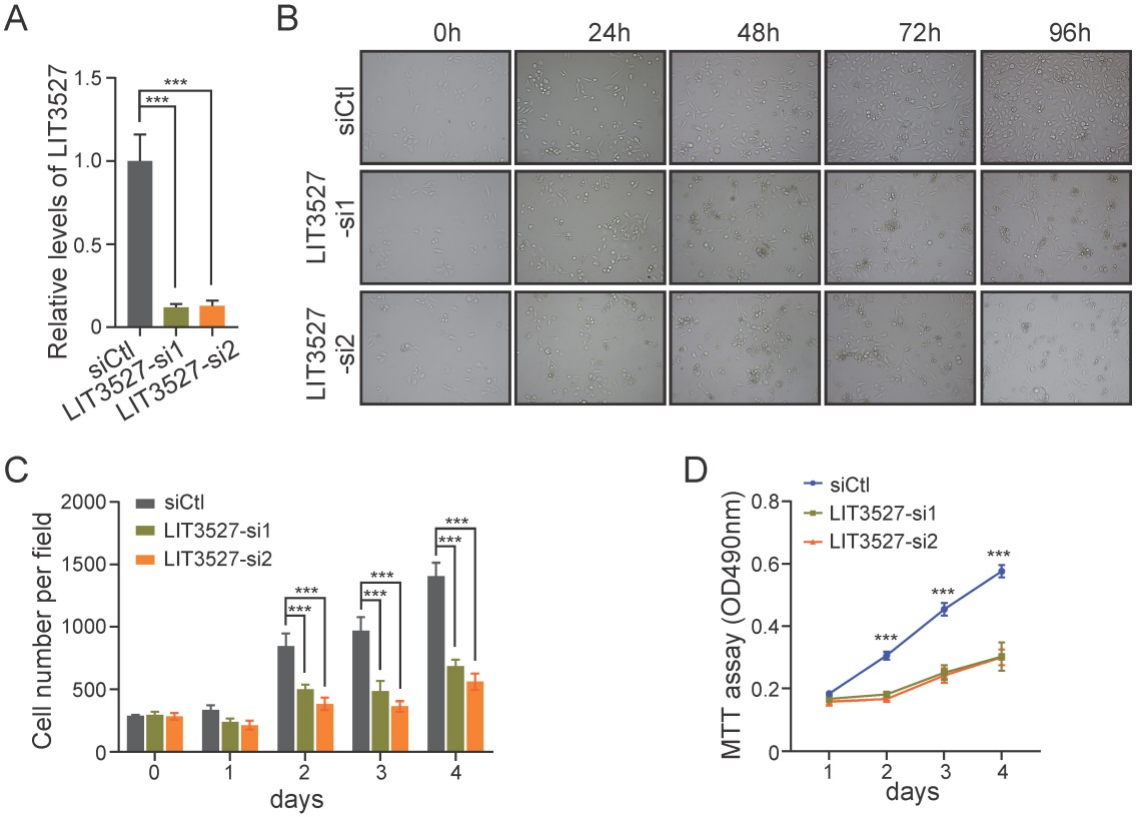
** LIT3527 is essential for gastric cancer cell proliferation. (A)** The expression levels of LIT3527 in AGS cells transfected with scrambled siRNA control, or two different siRNAs targeting LIT3527. **(B and C)** AGS cells transfected with indicated siRNAs were examined at the indicated time points. Representative views were shown (B). The cell number per field (n = 6) was counted (C). **(D)** MTT assay were performed to detect the cell proliferation of BGC-823 cells transfected with scrambled siRNA control, or two different siRNAs targeting LIT3527. Data are shown as mean ± SD. ***P < 0.001; Student's *t* test (*A*, *C*, *D*).

To further investigate the biological functions of LIT3527 in gastric cancer progression, we designed two different siRNAs against LIT3527. LIT3527 was dramatically silenced in AGS cells (Fig. 2A). We observed that AGS cells transfected with LIT3527-siRNAs displayed notable inhibition of cell proliferation as shown by bright field photographs (Fig. 2B) and the cell number counting at indicated time points (Fig. 2C). We also knocked down LIT3527 in BGC823 cells and performed MTT assays. Similar results were observed that knockdown of LIT3527 expression significantly inhibited the cell proliferation of BGC823 cells (Fig. 2D). These results indicate that LIT3527 is essential for gastric cancer cell proliferation.

### Knockdown of LIT3527 inhibits cell migration of gastric cancer cells

**Figure 3:**
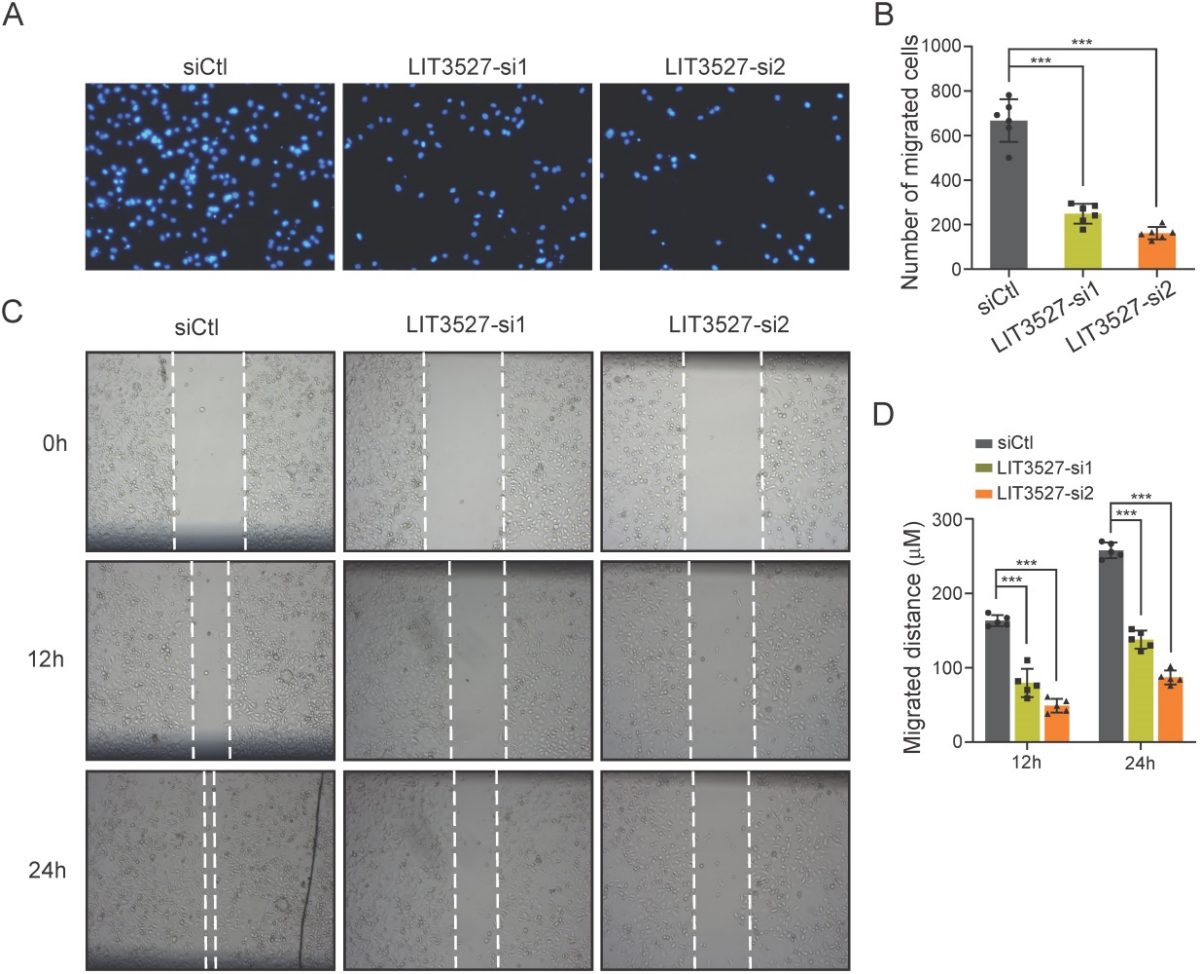
** Knockdown of LIT3527 inhibits cell migration of gastric cancer cells. (A and B)** Transwell assays were performed to examine the cell migration of gastric cancer cells. AGS cells were transfected with scrambled siRNA control, or two different siRNAs targeting LIT3527. Cells were seeded in the upper chambers. AGS cells were allowed to migrate for 18h. The migrated cell were stained with DAPI and photographed. The representative views were shown (A). The number of migrated cells were counted (B). **(C and D)** The AGS cells transfected with indicated siRNAs were subjected to wound healing assay. The scratch distances were measured at the 12h and 24h. The representative views were shown (C). The migrated distance were quantified (D). Data are shown as mean ± SD. ***P < 0.001; Student's *t* test (*B*, *D*).

Next, we tried to investigate the role of LIT3527 in the cell migration of gastric cancer cells. AGS cells transfected with siRNAs against LIT3527, or scrambled siRNA were subjected to transwell assay. Migrated cells were stained with DAPI and counted. Transwell assays showed that depletion of LIT3527 dramatically inhibited the cell migration of AGS cells (Fig. 3A, B). We further performed scratch wound healing assay. In agreement with the results from transwell assay, the migrated distances of the AGS cells with depleted LIT3527 were significantly shorter than the control AGS cells (Fig. 3C, D). These results suggest that knockdown of LIT3527 inhibits the cell migration of gastric cancer cells.

### Knockdown of LIT3527 induces cell death of gastric cancer cells

**Figure 4:**
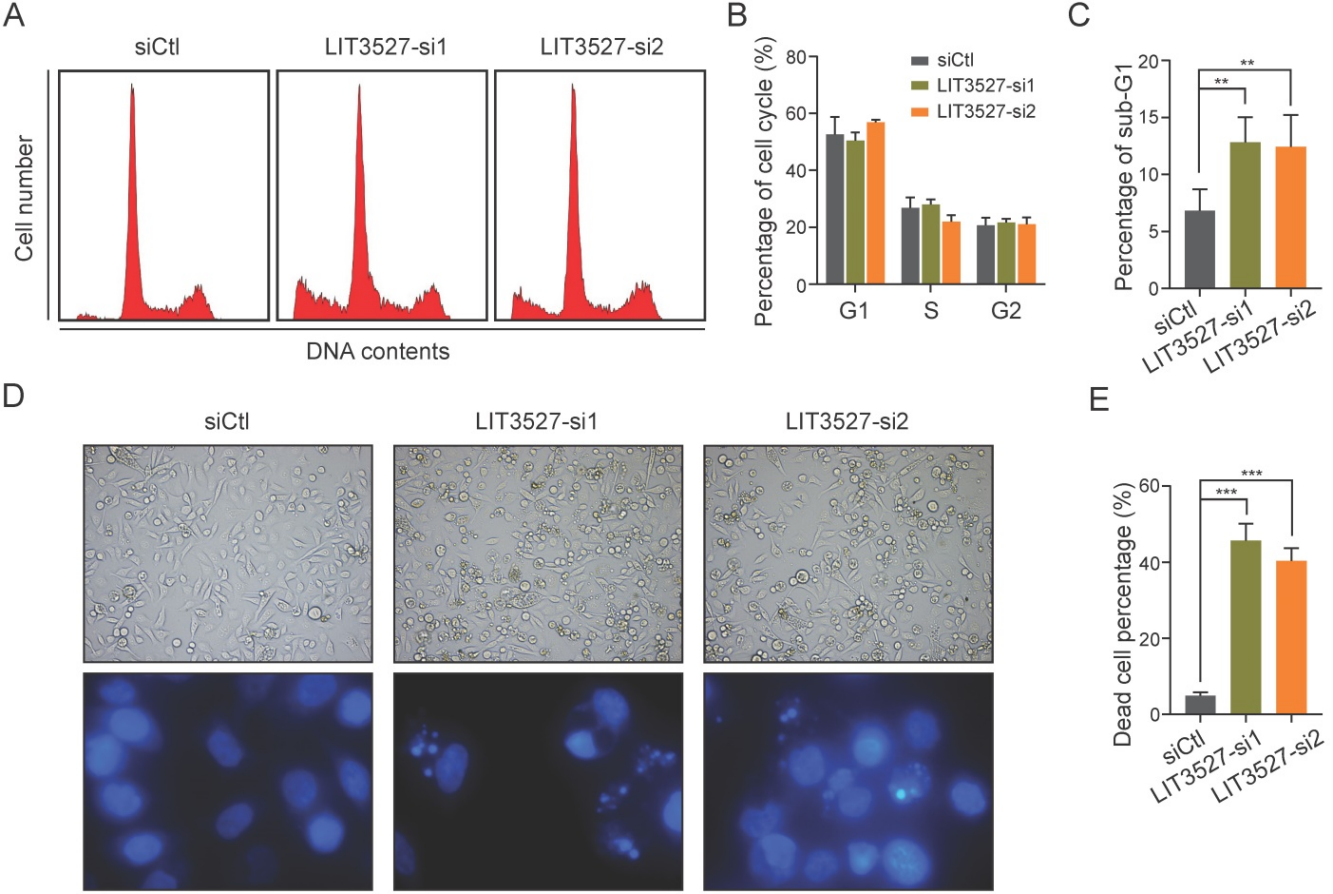
** Knockdown of LIT3527 induces cell death of gastric cancer cells. (A-C)** AGS cells transfected with indicated siRNAs were subjected to cell cycle analysis by FACS assay. Representative result was shown (A). The cell cycle phases were quantified (B). The sub-G1 phase was quantified (C). Knockdown of LIT3527 induces cell death of AGS cells. **(D and E)** The indicated AGS cells were photographed and stained with DAPI. Representative views showing the increased cell death (F). The death cell percentage was quantified (G). Data are shown as mean ± SD. **P < 0.01, ***P < 0.001; Student's *t* test (*C*, *E*).

We tried to explore the possible mechanisms underlying the effects of LIT3527 on cell proliferation and migration. We applied flow cytometry analysis to examine cell cycle and found that depletion of LIT3527 in AGS cells had no significant effect on cell cycle (Fig. 4A, B). Interestingly, we observed a notable increase in the percentage of sub-G1 phase, suggesting that knockdown of LIT3527 induced cell death of gastric cancer cells (Fig. 4C). Both bright field photographs and DAPI staining displayed the severe cell death induced by depletion of LIT3527 (Fig. 4D, E). These results suggest that LIT3527 is essential for gastric cancer cell survival but not cell cycle.

### Knockdown of LIT3527 induces apoptosis and autophagy of gastric cancer cells

**Figure 5:**
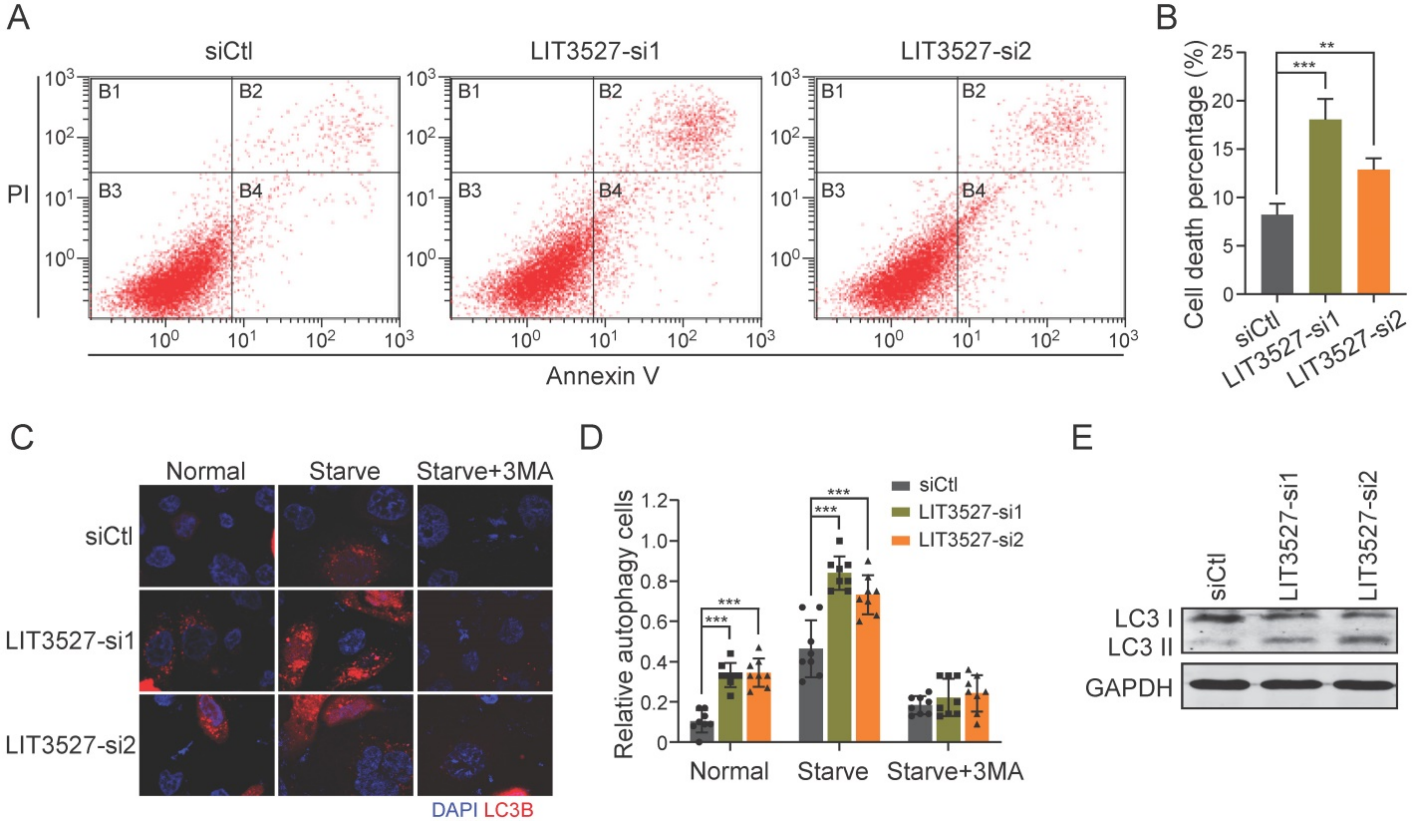
** Knockdown of LIT3527 induces apoptosis and autophagy of gastric cancer cells. (A and B)** AGS cells transfected with indicated siRNAs were applied for flow cytometry analysis with FITC-conjugated annexin V and propidium iodide staining. Knockdown of LIT3527 induces cell apoptosis of AGS cells. **(C and D)** Immunofluorescence analysis of cell autophagy with LC3 antibody. AGS cells transfected with indicated siRNAs were starved with serum for 24h or not. 3-MA is a specific inhibitor of cell autophagy. Representative views were shown (C). The percentage of LC3-positive cells was quantified (D). **(E)** The indicated AGS cells were subjected to western blot analysis. Antibodies against LC3, GAPDH were used. Data are shown as mean ± SD. **P < 0.01, ***P < 0.001; Student's *t* test (*B*, *D*).

To understand the roles of LIT3527 in cell survival, we stained AGS cells with annexin V and propidium iodide. Flow cytometry analysis showed that depletion of LIT3527 significantly induced cell apoptosis of AGS cells (Fig. 5A, B). Next, we tried to investigate whether knockdown of LIT3527 will induce autophagy of gastric cancer cells. Immunofluorescence showed that AGS cells transfected with siRNAs targeting LIT3527 displayed high levels of LC3 puncta (Fig. 5C, D). Depletion of LIT3527 also increased serum starve-induced autophagy of AGS cells (Fig. 5C, D). The effects can be blocked by 3-methyladenine (3-MA) treatment, a specific inhibitor of autophagy (Fig. 5C, D). In addition, western blot analysis also confirmed the upregulation of LC3-II levels by silence of LIT3527 (Fig. 5E). Taken together, these results suggest that depletion of LIT3527 induces apoptosis and autophagy of gastric cancer cells.

### LIT3527 regulates mTOR signaling

**Figure 6:**
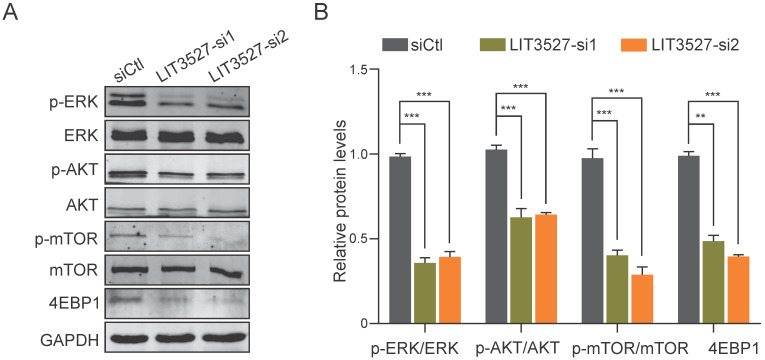
** LIT3527 regulates mTOR signaling. (A)** The indicated AGS cells were subjected to western blot analysis. Antibodies against phosphorylated ERK, ERK, AKT, phosphorylated mTOR, mTOR, 4EBP1, GAPDH were used. **(B)** The intensity of immunoblot band was measured by Image J software. The immunoblot assay was repeated three times. Data are shown as mean ± SD. **P < 0.01, ***P < 0.001; Student's *t* test (*B*).

Considering the functions of LIT3527 in apoptosis and autophagy of gastric cancer cells, we analyzed several pathways and found that mTOR pathway was involved. Western blot analysis showed that depletion of LIT3527 dramatically decreased the phosphorylation of mTOR and its downstream 4EBP1 (Fig. 6A, B). AKT and ERK, as the upstream of mTOR, were also obviously inhibited by knockdown of LIT3527 (Fig. 6A, B). These results suggest that LIT3527 regulates cell survival of gastric cancer cells may through modulating mTOR signaling.

### Knockdown of LIT3527 suppresses metastasis of gastric cancer cells

**Figure 7:**
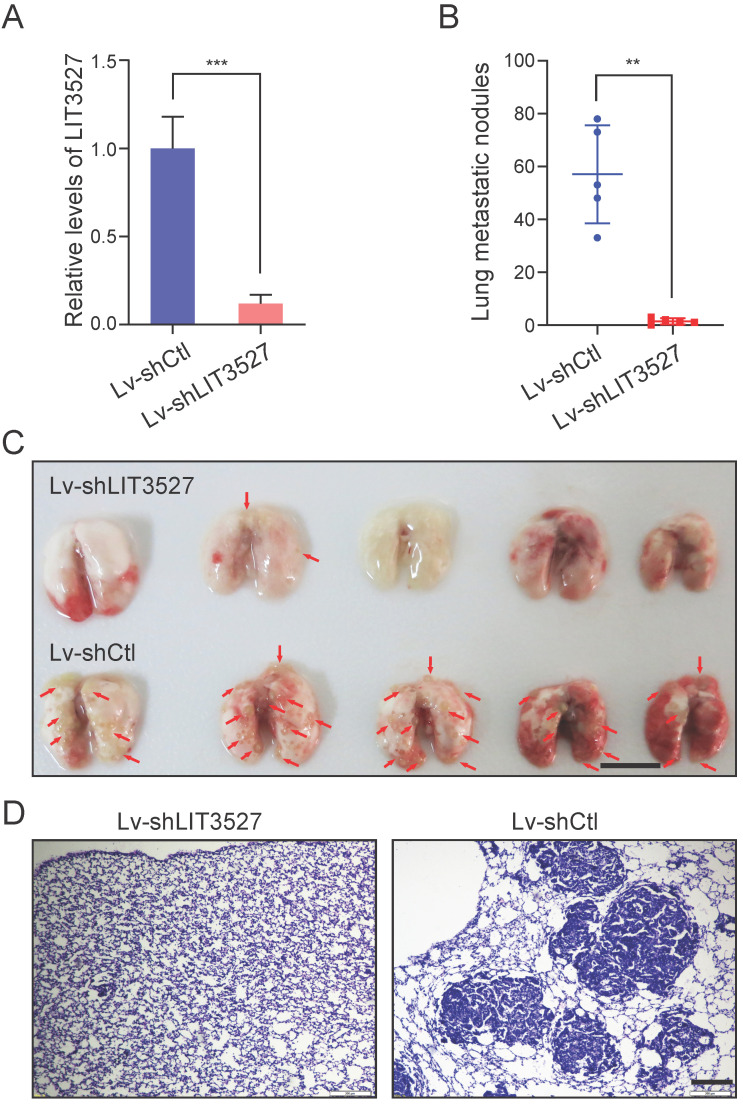
** Knockdown of LIT3527 suppresses metastasis of gastric cancer cells. (A)** BGC-823 cells were transfected with the lentivirus-based shRNA targeting LIT3527 or control shRNA, and subjected to qRT-PCR analysis. **(B and C)** LIT3527-depleted BGC-823 cells were intravenously injected into nude mice (n = 5). Mice were sacrificed 5 weeks after inoculation. The lung metastasis nodules were counted (B). The gross lung were photographed (C). Red arrows indicate lung metastasis lesions. **(D)** The lung tissues were subjected to H & E staining. Representative views showing the metastatic lesions. Data are shown as mean ± SD. **P < 0.01, *** P < 0.001. Student *t* test (*A*), Mann-Whitney test (*B*). Scale bar, 1 cm (*C*); 200 µm (*D*).

The crucial roles of LIT3527 in cell survival and migration of gastric cancer cells promoted us to investigate the function of LIT3527 in gastric cancer cell metastasis *in vivo*. BGC-823 cell line were employed because of its malignancy for *in vivo* metastasis 11. We established LIT3527-depleted BGC-823 by lentivirus-based shRNAs (Fig. 7A). BGC-823 cells were intravenously injected into nude mice. Mice received control BGC-823 cells developed severe lung metastatic nodules (Fig. 7B, C). Depletion of LIT3527 dramatically inhibited lung metastasis of BGC-823 cells (Fig. 7B, C). H & E staining of lung sections confirmed the results (Fig. 7D).

## Discussion

Gastric cancer has become the second leading cause of cancer-related mortality. Due to the lack of effective therapy, successful clinical management of metastatic GC remains a major challenge. Elucidating the pathogenesis and molecular mechanisms of gastric cancer are important for development of effective therapeutic strategies against gastric cancer. LncRNAs have been extensively studied in various cancers and were involved in tumorigenesis and progression of cancers, including gastric cancer 21. Here, we identified a new gastric cancer-related lncRNA LIT3527 that was frequently upregulated in gastric cancer. LIT3527 was essential for gastric cancer cell survival. Knockdown of LIT3527 induced severe apoptosis and autophagy, leading to cell death. Mechanistically, we uncovered that LIT3527 may be crucial for maintaining activity of mTOR pathway.

Apoptosis is activated by extrinsic or intrinsic inducers, playing an anticancer role. It has been well documented that many lncRNAs regulated gastric cancer malignancy through affecting apoptosis. LncRNA TINCR could bind to STAU1, influenced KLF2 mRNA stability, and regulated CDKN1A/p21 expression, thereby affecting proliferation and apoptosis of gastric cancer cells 22. LINC01234 functioned as a ceRNA for miR-204-5p to upregulate target CNFB. Knockdown of LINC01234 induced apoptosis and *in vitro* and inhibited tumorigenesis *in vivo* 23. LncRNA CASC9 could interact with BMI1 and upregulated BMI1 expression. Silencing CASC9 promoted apoptosis of gastric cancer cells 24. Silencing of lncRNA MALAT1 suppressed cell proliferation and promoted apoptosis of gastric cancer cells through increasing miR-22-3p and decreasing ErbB3 25. LncRNA DCST1-AS1 was highly expressed in gastric cancer tissues. Depletion of DCST1 inhibited cell proliferation and promoted cell apoptosis in gastric cancer cells through inhibition of miR-605-3p 26. In this study, we characterized LIT3527 as a survival-related gene. Knockdown of LIT3527 induced notable apoptosis and autophagy of gastric cancer cells.

Cell autophagy is characterized by the degradation of cellular organelles, which was considered as an energy source. It plays critical role for maintaining cellular homeostasis 27. Therefore, although autophagy is one of the two main types of programmed cell death, it functions as both an oncogenic and ant-tumorigenic process in cancer 10. During the autophagy process, cytosolic LC3 (LC3-I) was transformed to LC3- phosphatidylethanolamine conjugate (LC3-II), which was recruited to autophagosomal membrane. Therefore, the autophagosomal marker LC3-II can reflect the autophagy activity 28. In this study, we found that depletion of LIT3527 induced significantly increased LC3-II/LC3 levels and LC3 puncta, suggesting that LIT3527 is involved in modulating gastric cancer cell autophagy. Although many lncRNAs have been reported to regulate cell proliferation or apoptosis, little is known about the role of lncRNAs in autophagy of gastric cancer cells. LncRNA JPX enhanced gastric cancer malignancy by regulating CXCR6 and autophagy via blocking miR‑197 29. Knockdown of lncRNA CCAT2 inhibited cell viability and cell cycle, and promoted the apoptosis and autophagy of gastric cancer cells may though blocking mTOR signaling 30. Another research identified a gastric cancer related-lncRNA HAGLROS, which modulated mTOR activity through sponging miR-100-5p, thereby promoted cell proliferation, migration and inhibited autophagy 31.

The mTOR pathway integrates both intracellular and extracellular signals and acts a pivotal role in cell homeostasis, including cell metabolism, cell growth and survival through phosphorylation of its downstream substrates that include 4E-binding protein 1 (4EBP1), S6 kinase (S6K) and others 32. Therefore, mTOR pathway was considered as a therapeutic target for cancer, including gastric cancer 33. Several studies have reported that high levels of phosphorylated mTOR were correlated with advanced tumor stage and unfavorable survival 34, 35. Everolimus, as an mTOR inhibitor used in clinic, is active and well-tolerated in gastric cancer patients with chemotherapy-refractory metastasis 36. LncRNAs were also involved in modulating mTOR pathway in gastric cancer. LncRNA NORAD inhibited apoptosis and promoted proliferation of gastric cancer cells by inhibiting miR-214 expression and activating Akt/mTOR pathway 37. LncRNA HAGLROS upregulated mTOR expression through sponging miR-100-5p. On the other hand, HAGLROS can bind with mTORC1 components to activate mTOR pathway, thereby maintained gastric cancer malignancy 31. Here, we found that knockdown of LIT3527 obviously suppressed phosphorylation of mTOR and downstream substrate 4EBP1. AKT and ERK, the two major upstream of mTOR, were also inhibited by depletion of LIT3527, suggesting that LIT3527 is involved in maintaining activity of mTOR pathway. It is of great interest to investigate how LIT3527 regulating mTOR pathway in future.

On the one hand, we provide evidences that LIT3527 is essential for the cell proliferation and cell survival of gastric cancer cells. On the other hand, the *in vitro* and *in vivo* data indicate that LIT3527 plays important role in gastric cancer cell migration and metastasis. mTOR signaling is well known for its roles in cell proliferation and cell survival. However, many studies have documented that mTOR signaling was involved in cell migration and metastasis. LncRNA MetaLnc9 promoted lung cancer metastasis through activation of AKT/mTOR signaling 38. Inhibition of dipeptidyl peptidase (DPP)-4 stimulated CXCL12/CXCR4 expression and activated mTOR signaling, which further induced epithelial-mesenchymal transition of breast cancer cells and metastasis 39. It is possible that LIT3527 regulates gastric cancer cell survival and migration through modulating mTOR signaling. As we known, metastasis is the leading cause of cancer-related death. Considering the important role of LIT3527 in the gastric cancer metastasis, it is deserved to further evaluate the potential significance of LIT3527 as a prognostic marker for gastric cancer metastasis.

In summary, the present study characterized LIT3527 as a gastric cancer cell survival-related gene. Targeting LIT3527 showed a dramatic inhibition of gastric cancer cell malignancy *in vitro* and metastasis *in vivo*. These results provide a new therapeutic strategy targeting lncRNA for gastric cancer patients.
